# ANZAED practice and training standards for mental health professionals providing eating disorder treatment

**DOI:** 10.1186/s40337-020-00333-0

**Published:** 2020-11-02

**Authors:** Kim Hurst, Gabriella Heruc, Chris Thornton, Jeremy Freeman, Anthea Fursland, Rachel Knight, Marion Roberts, Beth Shelton, Andrew Wallis, Tracey Wade

**Affiliations:** 1 Australia & New Zealand Academy for Eating Disorders, Sydney, Australia; 2 Eating Disorder Service, Robina Private Hospital, Robina, Australia; 3 School of Psychology, Griffith University, Gold Coast, Australia; 4 School of Medicine, Western Sydney University, Campbelltown, Australia; 5 Eating Disorder Service, Northern Sydney Local Health District, Sydney, Australia; 6 The Redleaf Practice, Sydney, Australia; 7 Western Australia Eating Disorders Outreach & Consultation Service, Nedlands, Australia; 8 Occupational Therapy, School of Health and Social Development, Faculty of Health, Deakin University, Geelong, Australia; 9 The Victorian Centre of Excellence in Eating Disorders, Melbourne, Australia; 10 Faculty of Medical & Health Sciences, University of Auckland, Auckland, New Zealand; 11 National Eating Disorders Collaboration, Melbourne, Australia; 12 Eating Disorder Service, Sydney Children’s Hospital Network, Sydney, Australia; 13 Blackbird Initiative, Órama Institute, Flinders University, Adelaide, Australia

**Keywords:** Eating disorders, Mental health clinician, Occupational therapist, Practice standards, Psychologist, Social worker, Mental health nurse, General practitioner, Training, Treatment

## Abstract

**Introduction:**

The Australia & New Zealand Academy for Eating Disorders (ANZAED) recently developed general principles and clinical practice standards recommended for mental health clinicians and dietitians providing treatment for people with eating disorders. Separate mental health practice and training standards were then devised as a foundation for strengthening the workforce and providing guidance to professional training programs and service providers on the minimal standards required for practice in the eating disorder field.

**Recommendations:**

The present recommendations for mental health professionals providing eating disorder treatment describe the following practice and training standards: eating disorder treatment foundations (including co-ordination of services, establishing a positive therapeutic alliance, professional responsibility and knowledge of levels of care), assessment, diagnosis, intervention (including evidence-based intervention, managing psychiatric risk and managing co-morbid mental health problems), and monitoring and evaluation.

**Conclusions:**

Further work is required to disseminate these standards to clinicians providing services across Australia to people with eating disorders, and to support adherence in the clinic room where they can translate to improved outcomes for clients. Pathways to supporting adherence include expert supervision of practice, incorporation in training and supervised practice in university settings, and support with checklists that can be used by consumers and referring professionals.

## Definition

Mental health clinicians help treat and diagnose individuals who have (or may have) any psychological issues.

Mental health professional includes many types of health professionals working in mental health care, including psychologists, social workers, mental health nurses, occupational therapists and counsellors.

## Plain English summary

The Australia & New Zealand Academy for Eating Disorders has developed clinical practice and training standards for mental health professionals on the treatment of eating disorders.

The clinical practice and training standards, based on published evidence and clinical experience were developed simultaneously with profession-specific practice and training standards (mental health and dietetics), which were reviewed and refined by each respective working group, and expert advisors. This was then split into three documents. The first documented general clinical practice and training standards for mental health professionals and dietitians providing eating disorder treatment [1]. The second outlined dietetic-specific practice and training standards [2]. This paper outlines the third document, the mental health-specific clinical practice and training standards. Eating disorder treatment foundations are covered including co-ordination of services, establishing a positive therapeutic alliance, professional responsibility and knowledge of levels of care. We then outline components of assessment, diagnosis, intervention, and monitoring and evaluation. Finally, training components for evidence-based psychotherapy practice and translating training into practice are described.

## Introduction

Although eating disorders are common, they are often underdiagnosed [3] and help-seeking may be more likely for co-occurring issues, such as anxiety and depression [4]. Improving the competencies of mental health professionals in early detection and treatment of eating disorders will make a significant difference to closing the well-recognised research-practice gap between what is identified as effective treatment and the actual treatment that patients receive [5]. Up to 40 % of psychologists fail to detect the presence of an eating disorder, and despite the significant medical complications of eating disorders, psychologists do not generally prioritise medical risk and management [6]. Reasons for potential deficits that may contribute to the low detection and treatment rates for eating disorders that have been identified in the literature include inadequate practitioner knowledge, skills and understanding [7–9].

Given the urgent need for early intervention for eating disorders, it is important that mental health clinicians feel confident to assess and treat patients. However, a clear system for determining competence to provide clinical treatment in the eating disorder field has not yet been defined. The Australia & New Zealand Academy for Eating Disorders (ANZAED) general clinical practice and training guidelines [1] represent an important first step toward the dissemination and implementation of evidence-based treatments into clinical practice. Their general aim is to inform clinical decision-making of healthcare professionals and patients about efficacious interventions and treatment strategies. The second step requires specific clinical practice and training standards to be developed for those clinicians offering psychotherapy for eating disorders, and this is the focus of the current work described herein.

### Methods of mental health-specific practice and training standards development

ANZAED brought together an expert group of clinicians and academics (nine mental health and eight dietetic professionals) from around Australia and New Zealand. The members all had greater than ten years’ experience within the field of eating disorders. The mental health professionals had backgrounds including psychology, social work and occupational therapy.

The clinical practice and training standards, based on published evidence and clinical experience were developed simultaneously with profession-specific practice and training standards (mental health and dietetics), which were reviewed and refined by each respective working group, expert advisors and ANZAED’s Executive Committee. These were then further reviewed, first at the ANZAED 2019 Conference in Adelaide via a public face-to-face consultation workshop (*n* = 100) without anonymity, and second by the broader public, via online consultation. In this way, feedback was received from international expert advisors, professional bodies (various disciplines) and consumer and carer groups with comments reviewed and integrated by the working group. The National Eating Disorders Collaboration (NEDC) Steering Committee and ANZAED’s Executive Committee reviewed the resulting document, which was then split into three documents. The first documented general clinical practice and training standards for mental health professionals and dietitians providing eating disorder treatment [1]. The second outlined dietetic-specific practice and training standards [2]. This paper outlines the third document, the mental health-specific clinical practice and training standards. Consensus on all papers was reached through discussion by the authors and there was no identified conflict of interest.

### Mental health-specific clinical practice standards

In addition to having competency in the ANZAED general clinical practice standards [1], it is recommended that mental health clinicians working with patients with eating disorders are competent in the mental health-specific clinical practice standards discussed below and summarised in Table 1.

**Table 1:** Mental health-specific clinical practice standards

1. **Co-ordination of services**	
1.1. **Communication within the multidisciplinary care team**	
i. Understand the significance and importance of a multidisciplinary team in treatment, and know the role of each member	
ii. Know how to set up a multidisciplinary team consistent with the treatment model being delivered	
iii. Participate in implementing treatment recommendations consistent with own professional background and work collaboratively with other team members both within and external to own practice	
iv. Understand the impact eating disorder pathology can have on the functioning of the multidisciplinary team (e.g.) The therapeutic process can be greatly impeded by incidents of splitting	
v. Ensure processes and systems are set up for effective communication between all parties, such as sharing case notes, letters, case conferencing, to ensure consistent, collaborative care	
1.2. **Communication with patients**	
i. Explain the limits of confidentiality and establish treatment non-negotiables	
ii. Outline an individual case formulation and explain how treatment goals relate to this formulation	
1.3. **Communication with family and significant others**	
i. Understand the role of families and carers in assessment, engagement, treatment and recovery support for children, young people and adults	
ii. Work collaboratively, and in an age appropriate way, with patients to allow their family to share information with the treatment team	
iii. If divisions around key issues and concerns arise within the family, ensure these are discussed openly, whilst providing understanding and support to the patient, and resolving these in favour of reducing the impact of the eating disorder	
2. **Establishing a positive therapeutic alliance**	
i. Recognise the central role the therapeutic alliance plays in therapy and the importance of the therapeutic alliance in the variability of patient outcome	
ii. Work to develop a relationship where the patient feels heard and respected.	
iii. Outline the tasks of therapy consistent with the therapist’s model of working. (e.g.) outlining the core principles of a cognitive behavioural therapy.	
iv. Obtain a collaborative and informed agreement about the tasks and goals of therapy. (e.g.) highlighting the role of dietary restriction in anorexia and binge eating and working collaboratively with the patient to obtain an understanding that reversing dietary restriction is an active goal of therapy	
v. Understand that there is a complex bidirectional interplay between therapeutic alliance, readiness for change, self-efficacy and early behaviour change.	
vi. Wherever possible early behaviour change should be the initial aim of therapy as it has shown to be correlated to better therapeutic alliance and better outcomes	
3. **Professional responsibility**	
i. Understand the need for a developmentally sensitive family and person-centred approach to setting up and implementing treatment	
ii. Understand the impact of culture, mental health stigma, weight bias and stigma that can prevent people from accessing support	
iii. Consistently reflect on and judge knowledge and experience limitations so that safe care is consistently provided	
iv. Understand the importance of clinical supervision as required by own professional body to enable effective reflective practice, as well as to support treatment planning and therapy model implementation	
v. Understand that clinical supervision on eating disorder cases should be undertaken with a clinician experienced in eating disorders and that regular eating disorder focused clinical supervision is an essential professional requirement. In addition, clinical supervision supports clinicians to know the limits of their expertise and when to seek advice or refer on	
vi. Understand the importance of continuing professional development as required by their professional body with the aim to develop the knowledge, skills and attitudes required to provide and manage mental health care for people experiencing an eating disorder	
4. **Knowledge of levels of care**	
i. Understand the services that are available at different care levels locally and match the treatment context to symptom severity	
ii. Understand how the treatment context will impact the implementation of an evidence-based model	
iii. Recognise the indicators for referral to a higher level of care (e.g. as an inpatient or day patient) and the aim of each care level. Factors like patient age and illness severity and duration as well as available access to higher levels of care will impact on decision making	
iv. Understand when and how involuntary treatment is implemented for the person with an eating disorder as a matter of urgency and duty of care	
v. Determine when to refer on for further assessments to address physical, psychiatric or nutritional needs	
5. **Mental health assessment**	
i. Know how to assess eating disorder symptoms, being aware that symptoms may be minimised by the person with an eating disorder • Bingeing, purging and compensatory behaviour ▪ Type of compensatory behaviour (e.g. laxative use, excessive exercise, diet pills, steroid use) ▪ Frequency ▪ Amount ▪ Types of food ▪ Triggers to binge • Height, weight and rate of any weight changes • Core cognitive features ▪ Over evaluation of weight and shape ▪ Eating-related cognitions (e.g. guilt, control) ▪ Body dissatisfaction ▪ Body checking ▪ Fear of fatness and weight gain ▪ Perfectionism • Food intake • Eating behaviours ▪ Past and current, and motivation to change these ▪ Food rituals ▪ Avoided foods and food sensitivities ▪ Fluid intake • Medical consequences of disordered eating behaviours • Psychosexual and interpersonal functioning • Treatment history • Comorbidity (medical and psychological) and be aware of the impact of nutritional status on mood and anxiety • Mental state assessment • Mental health risk factors (e.g. suicidality) • Family of origin and support system • Trauma history • Psychometric assessment – such as the Eating Disorder Examination-Questionnaire (EDE-Q) or the ED-15. The ED-15 and the 12-item EDE-Q are suitable for a session by session assessment of progress, which is also shown to enhance the effectiveness of treatment.	
ii. Understand the common medical co-occurring diagnoses that are typical for people with an eating disorder and assess the short- and long-term physical impacts of an eating disorder, including the need for urgent assessment and intervention if there is a risk of medical instability	
iii. Understand the common psychiatric co-occurring diagnoses that are typical for people with an eating disorder; including risk of suicide and self-harm	
iv. Assess the range of impacts an eating disorder has across the domains of life; ascertain illness progression and its impact on psychological, social and quality of life factors	
v. Identify and support the recognition of strengths and resources for the person with an eating disorder	
vi. Know how to engage key parents, carers or significant others	
vii. Understand the use of and implementation of age-appropriate validated eating disorder assessment tools and psychometric tests	
6. **Mental health diagnosis**	
i. Ability to identify the diagnostic criteria for eating disorders, and apply appropriately including distinguishing from differential diagnoses	
ii. Understand the risk factors that contribute to the development of eating disorder, including awareness of populations at high risk for developing at eating disorder	
iii. Understand the signs of an eating disorder at different stages of progression and have an awareness of the way in which the seriousness of eating disorder symptoms can be minimised when clinicians are poorly informed	
iv. Identify when a person requires urgent medical assessment or psychiatric assessment and when they should be referred to a hospital emergency department	
v. Develop a case formulation including preliminary hypotheses about predisposing, precipitating and maintaining factors, as well as noting the individual’s strengths and protective factors	
7. **Mental health intervention**	
**7.1 Knowledge of evidence-based treatment**	
i. Understand, describe and have a working knowledge of the current evidence base for eating disorder treatments and how they are implemented for each diagnosis • Weighing of the patient at each appointment and sharing that information with the client wherever possible • Reviewing eating and disordered behaviours • Establishing regular eating • Identifying antecedent variables (triggers) to eating disorder behaviour • Working with families or significant others to support the patient towards recovery, in a developmentally appropriate way. For children or adolescents this will include eating disorder-focussed family therapy. • Provision of alternative coping strategies to replace the eating disorder. This is likely to involve seeking support and strategies to manage affect and relational triggers • On an ongoing basis, addressing issues of motivation, body image and quality of life	
ii. Provide support to a person with an eating disorder and their family and significant other (where possible) while awaiting specialist care or evidence-based treatment to commence	
iii. Have awareness of components of effective medical management and ensure that regular medical reviews are occurring	
iv. Understand how the symptoms of an eating disorder can impact engagement and adherence to treatment; it is important to have clear treatment goals and outcomes to mitigate against poor progress	
v. Put risk management plans in place for self-harm and suicidal ideation	
vi. Have a knowledge of developmentally appropriate basic nutritional and healthy eating principles	
vii. Advise the person and family or significant other (where possible) about basic immediate steps and their benefits such as regular eating, supervision and support, the need for weight gain	
viii. Understand the complex relationships between motivation and behavioural change for people with eating disorders and that ambivalence is common in eating disorder presentations, despite a strong underlying need to feel better	
ix. Understand the need for speciality training and supervision to learn and implement a treatment model competently and with fidelity	
8. **Managing risk**	
i. Identify and manage suicidality at all levels of eating disorder care. Risk factors for suicide can include: • previous suicide attempts • deliberate self-harm • substance abuse • comorbid depression • additional mental illness • social isolation • lack of fear of death • access to medication and others means of harm • increased family conflict • thinking that they are a burden • impulsive traits • experiences of trauma (including but not limited to post-traumatic stress disorder) severity of eating disorder • recent rapid weight loss	
ii. Identify protective factors	
iii. Manage co-occurring mental health diagnoses across the course of treatment	
9. **Monitoring and evaluation**	
i. Measure treatment adherence and outcomes using methods that are standardised or of an accepted standard in the field such as monitoring weight, binge-purge frequency, or eating disorder psychopathology with psychometric measures	
ii. Provide a follow-up schedule that matches the severity of the eating disorder and the treatment model being implemented	

#### Eating disorder treatment foundations

##### Co-ordination of services

The mental health clinician needs to understand the significance, importance and role of a multidisciplinary team in treatment. They must also work collaboratively and consistently within their professional scope of practice and ensure effective communication between all parties. The limits of confidentiality need to be explained and the mental health clinician should join with the patient and family (where possible) to establish treatment non-negotiables.

##### Establishing a positive therapeutic alliance

It is essential that the mental health clinician is able to establish and maintain a positive therapeutic alliance with the patient and significant others. Components of the therapeutic alliance include: i) a strong therapeutic relationship where the patient feels heard, understood and respected; ii) an agreement of the approach or method of treatment where there is a goodness of fit between therapist approaches and patient expectations and iii) an agreement on the goals and topics of therapy (e.g., establishing regular eating) [10, 11]. The mental health clinician also needs to be aware of the complex interplay between therapeutic alliance, readiness for change, self-efficacy and early behaviour change. Early behaviour change should be the aim of therapy as it has been shown to improve the therapeutic alliance and is related to better outcome [12, 13].

##### Professional responsibility

The mental health clinician will understand the need for a developmentally sensitive family and person-centred approach to setting up and implementing treatment within the boundaries prescribed by their profession and codes of ethical practice. Of great importance is having an understanding of the impact of culture, mental health stigma and weight bias that can prevent people from accessing support. It is expected that the mental health clinician understands the importance of, and engages in, clinical supervision undertaken with a clinician experienced in eating disorders, this could be delivered by the same or different professional discipline to the clinician. Ongoing professional development is essential, with the aim to develop the knowledge, skills and attitudes required to provide and manage mental health care for people experiencing an eating disorder.

##### Knowledge of levels of care

The mental health clinician needs to understand the services that are available at different care levels locally and can match the treatment context to symptom severity. In addition, mental health clinicians should understand when and how involuntary treatment is implemented for the person with an eating disorder as a matter of urgency and duty of care.

#### Mental health assessment

A thorough assessment should confirm or refute a diagnosis or diagnoses, explore elements of risk and develop a case formulation, which informs the treatment plan and priorities. As well as information from a general assessment, such as demographics, an eating disorder assessment [14] should cover additional topics specific to eating disorder management.

The mental health clinician needs to know how to assess eating disorder symptoms; and should be aware of the impact of nutritional status on mood and anxiety; understand the common medical and psychiatric co-occurring diagnoses that are typical for people with an eating disorder; identify and support the recognition of strengths and resources for the person with an eating disorder and how to engage significant others in their care; and understand the use of and implementation of age-appropriate validated eating disorder assessment tools and psychometric tests.

Eating behaviours should be assessed in detail, including past and current eating patterns and motivation to change these, food rituals, avoided foods and food sensitivities and fluid intake. Beliefs that accompany these rituals should also be assessed. The core manifestations of the eating disorder include severe restriction of food intake, binge eating, compensatory behaviours (e.g., purging, laxative use, excessive exercise, diet pills, diuretics, steroid use), the frequency of these behaviours, the amount and type of food eaten and binge triggers. Height, weight and rate of any weight changes should be noted, as should weight history. The core cognitive features of the eating disorder should also be assessed, including over evaluation of weight and shape, eating-related cognitions (e.g. guilt, control), body dissatisfaction and associated body avoidance and checking, and fear of fatness and weight gain.

A number of psychometric assessments – such as the Eating Disorder Examination-Questionnaire (EDE-Q) [15] or the ED-15 [16] should routinely be administered. The ED-15 and the 12-item EDE-Q [17] are suitable for a session by session assessment of progress, which is also shown to enhance the effectiveness of treatment [18].

It is important to note any psychiatric comorbidity, including trauma history, self-harm and suicidality. A full mental state assessment should be undertaken as well as an assessment of the family of origin and support system. Further, psychosexual functioning, the patient’s treatment history and maintaining factors such as perfectionism, self-criticism and low self-esteem, mood intolerance and interpersonal functioning need to be explored.

In addition, the mental health clinician should understand the medical consequences of disordered eating behaviours, identify the need for a medical assessment and have an awareness of what is covered in such an assessment [19]. A medical assessment (e.g., following RANZCP guidelines [19] is different from a mental health assessment, and while identifying physical signs and symptoms of eating disorder is expected and needs to be communicated to the individual’s medical practitioner, providing a medical assessment and diagnoses is not within the mental health clinician’s scope of practice, unless medically qualified to do so. It is important, however, to identify when a person appears to require urgent medical assessment or psychiatric assessment and when they should be referred to a hospital emergency department.

#### Mental health diagnosis

The mental health clinician should possess the ability to identify the diagnostic criteria for eating disorders and make any differential diagnoses. The core manifestations identified through the mental health assessment will aid the eating disorder diagnosis. The clinician must also develop a case formulation including preliminary hypotheses about predisposing, precipitating and maintaining factors, as well as noting the individual’s strengths and protective factors. The case formulation should be based on the evidence-based treatment model being used by the clinician. The formulation should be collaboratively co-authored with the patient and significant others if relevant, and form the foundation of treatment.

#### Mental health intervention

##### Evidence-based intervention

Mental health clinicians should maintain an understanding of the current evidence-based treatments for eating disorders [20–22] and how they are implemented for each diagnosis. They must have appropriate training, as well as ongoing professional development and supervision in the implementation of these treatments. Components of effective treatment would usually include:

- Weighing of the patient at each appointment and sharing that information with them [23];
- Reviewing eating and disordered behaviours;
- Establishing regular eating;
- Eliminating restrictive eating;
- Identifying antecedent variables (triggers) to eating disorder behaviour;
- Working with families or significant others to support the patient towards recovery, in a developmentally appropriate way (for children or adolescents this will include eating disorder-focussed family therapy);
- Provision of alternative coping strategies to replace the eating disorder – this is likely to involve seeking support and strategies to manage affect and relational triggers;
- On an ongoing basis, addressing issues of motivation, body image and quality of life.

##### Managing psychiatric risk

Individuals with an eating disorder present with elevated rates of death by suicide compared to other mental health disorders (e.g., depression and schizophrenia). Therefore, identifying and managing suicidality is imperative at all levels of care. Risk factors for suicide [24] can include: previous suicide attempts; deliberate self-harm; substance abuse; comorbid depression; additional mental illness; social isolation; lack of fear of death; access to medication and others means of harm; increased family conflict; thinking that they are a burden; impulsive traits; experiences of trauma (including but not limited to post-traumatic stress disorder); severity of eating disorder and recent rapid weight loss. Protective factors (e.g. support networks, positive sense of identity and cultural heritage, effective coping and problem-solving skills) should also be identified by the mental health clinician. If psychiatric risk is identified, clinicians should liaise with the members of the multidisciplinary team to determine the appropriate course of action (e.g. review by psychiatrist or send the patient to the emergency department for assessment).

##### Managing comorbid mental health problems

Mental health clinicians treating patients with eating disorders need to also manage co-occurring mental health diagnoses across the course of treatment. This may include providing concurrent treatment or referring to another professional for follow up and management.

#### Monitoring and evaluation

An important component of monitoring is to include a measure of treatment adherence and outcomes using methods that are standardised or of an accepted standard in the field such as monitoring weight, binge-purge frequency, or eating disorder psychopathology with psychometric measures. Once therapy concludes, the mental health clinician should develop a follow-up plan that matches the severity of the current eating disorder presentation and the treatment model being implemented.

### Mental health-specific training standards

Mental health-specific clinical training should either partially or entirely address the mental health-specific clinical practice standards outlined above, depending on the duration and intensity of the training course. Training to achieve competency in these practice standards will occur via multiple pathways, including formal and informal learning opportunities. Components of training are summarised below and detailed in Tables 1 and 2. It is essential that mental health clinicians seeking training in a specific evidence-based model receive training at a level that meets the following content and quality standards.

**Table 2:** Training components for evidence-based psychotherapy practice

Workshop content	
Workshops on a specific evidence-based model should contain the following:	
• An up-to-date research review for the model’s inclusion as an evidence-based model in the relevant clinical practice guidelines	
• The core concepts of the model and its underlying theory	
• The structure and phases or stages of the treatment	
• Information consistent with the published manual if manualised and address issues of model efficacy	
• Recommendations on the implementation and application of the model in different practice settings and within a multidisciplinary team	
• A reflective component for clinicians to appreciate how they may need to change their practices to reflect the model being taught	
• A significant practice component in the form of video demonstration, role play, case scenarios or similar, to enable the translation of theory to practice	

It is essential that mental health clinicians seeking training in a specific evidence-based model receive training at a level that meets the following content and quality standards. Training in evidence-based models should extend on the foundational training outlined in Table 2

#### Training components for evidence-based psychotherapy practice

Training programs and workshops should have clear learning objectives and should be of a suitable length to meet the stated aims of the training. Additionally, the capacity of the training to provide the skills to use the model in practice after the training event should be clearly stated. Benchmarking training in specific eating disorder models against equivalent training internationally provides a good guideline for locally provided training. Institutions, training organisations and individuals providing training in specific models should be suitably qualified, recognised for their expertise in the model and have significant experience implementing the treatment in different practice settings. Where training in a specific eating disorder model is being implemented in a specific context, the institutions, training organisation or individual should have appropriate experience providing treatment to meet the needs of the context such as community, private practice or tertiary settings.

#### Translating training into practice

It is recognised that attending eating disorder training does not automatically translate to a suitable level of capacity to implement a model at the required level to provide treatment efficacy that will result in acceptable patient outcomes. Clinicians should recognise the need for post-training, model-specific eating disorder clinical supervision and additional professional development to develop the expertise to implement the model for individual patients and families. This could include but is not limited to advanced practice workshops, observing more experienced clinicians, professional reading programs, and conference workshops. This need should be integrated into their existing professional organisation continuing professional development requirements.

Consideration should also be given by a clinician to their previous experience in delivering eating disorders treatment, to determine what additional training is needed. New clinicians should proceed cautiously, recognising their need to develop both foundational as well as specific model-related skills. Model-specific supervision differs from general clinical supervision in that it should consider the developmental stage of the clinician in the model and thus provide a high ratio of teaching and instruction which will become more reflective over time. Supervision at the highest standard will involve the review of audio or video to support the clinician to develop appropriate micro-skills in the model. Clinicians learning a new model should plan for 1–2 years of supervised/supported practice (depending on their practice context) to become competent in a new model.

## Conclusion

The impact and consequences of an eating disorder can be minimised or avoided by early identification and management in the outpatient treatment setting [25]. Unfortunately, the research literature suggests that often mental health professionals feel ill equipped in this process [26]. Evidence suggests that adherence to some aspects of protocol that are perceived to be challenging (e.g., weighing patients in session) is low [27]. It is hoped that implementation of the practice and training standards described herein will promote a coordinated and consistent approach bringing treatment closer to best practice.

These mental health-specific clinical practice and training standards developed by ANZAED seek to ensure that mental health professionals meet safe standards to treat people with eating disorders. It is hoped that these standards will help professionals aim to achieve a level of competence in their skills and experience, resulting in improved patient outcomes. It is recognised, however, that training without expert supervision in psychotherapy does not necessarily result in changed behaviour [26, 28]. Thus, it is strongly recommended that these standards are utilised under regular expert supervision of practice.

The mental health-specific practice standards may also provide a useful framework from which universities and educational institutions can develop curriculum content. Given the requirement for provision of training clinics in Australian universities, this content can be best embedded by supervision of clinical practice in such settings. A recent review of supervisory practice in postgraduate psychology training, however, showed inconsistent use of evidence-based practice and variation in evidence-based supervision methods and techniques [29], such as use of competency evaluation rating forms. Evidence from Australian training clinics suggests that a combination of expert supervision and a clearly outlined protocol does ensure competency in the provision of therapy for individuals with an eating disorder, producing outcomes commensurate with therapies offered by expert clinicians [30].

It is also anticipated that these standards may form the basis of a credentialing system, which might aid general practitioners, people seeking treatment and their families to identify treatment providers who have the knowledge, training and experience to treat eating disorders safely and effectively. Other work to support these practice standards should be considered, such as development of accessible checklists for consumers within a co-design approach, as well as practice standards for general practitioners, psychiatrists and paediatricians who manage referrals for the treatment of serious eating disorders, and whose review of client progress is necessary for continuation of extended therapy.

## Data Availability

Not applicable.

## References

[1] Heruc G, Hurst K, Casey A, Fleming K, Freeman J, Fursland A, et al. ANZAED eating disorder treatment principles and general clinical practice and training standards. J Eat Disord. In press. 10.1186/s40337-020-00341-0 PMC7653831 33292546

[2] Heruc G, Hart S, Stiles G, Fleming K, Casey A, Sutherland F, et al. ANZAED practice and training standards for dietitians providing eating disorder treatment. J Eat Disord. In press. 10.1186/s40337-020-00334-z PMC7737344 33317617

[3] Fursland A, Watson HJ (2014). Eating disorders: a hidden phenomenon in outpatient mental health?. Int J Eat Disord..

[4] Hudson JI, Hiripi E, Pope HG, Kessler RC (2007). The prevalence and correlates of eating disorders in the national comorbidity survey replication. Bio Psych.

[5] Shafran R, Clark DM, Fairburn CG, Arntz A, Barlow DH, Ehlers A (2009). Mind the gap: improving the dissemination of CBT. Behav Res Ther.

[6] Worsfold KA, Sheffield JK (2018). Eating disorder mental health literacy: what do psychologists, naturopaths, and fitness instructors know?. Eat Disord.

[7] Currin L, Waller G, Schmidt U (2009). Primary care physicians' knowledge of and attitudes toward the eating disorders: do they affect clinical actions?. Int J Eat Disord..

[8] Jones W, Saeidi S, Morgan J (2013). Knowledge and attitudes of psychiatrists towards eating disorders. Euro Eat Disord Rev.

[9] Jorm AF (2000). Mental health literacy. Public knowledge and beliefs about mental disorders. Br J Psychiatry.

[10] Bordin ES (1979). The generalizability of the psychoanalytic concept of the working alliance psych: Theo. Res Prac.

[11] Wampold BE, Imel ZE (2015). Counseling and psychotherapy.The great psychotherapy debate: the evidence for what makes psychotherapy work (2nd ed.).

[12] Zaitsoff S, Pullmer R, Cyr M, Aime H (2015). The role of the therapeutic alliance in eating disorder treatment outcomes: a systematic review. Eat Disord.

[13] Graves TA, Tabri N, Thompson-Brenner H, Franko DL, Eddy KT, Bourion-Bedes S (2017). A meta-analysis of the relation between therapeutic alliance and treatment outcome in eating disorders. Intl J Eat Disord.

[14] Wade T, Pellizzer M, Sellbom M, Suhr J (2019). Assessment of eating disorders. Cambridge handbook of clinical assessment and diagnosis.

[15] Mond JM, Hay PJ, Rodgers B, Owen C, Beumont PJV (2004). Validity of the eating disorder examination questionnaire (EDE-Q) in screening for eating disorders in community samples. Beh Res Ther.

[16] Rodrigues T, Vaz AR, Silva C, Conceição E, Machado PPP (2019). Eating Disorder-15 (ED-15): factor structure, psychometric properties, and clinical validation. Eur Eat Disord Rev.

[17] Prnjak K, Mitchison D, Griffiths S, Mond J, Gideon N, Serpell L (2020). Further development of the 12-item EDE-QS: identifying a cut-off for screening purposes. BMC Psych.

[18] Vitousek K, Watson S, Wilson GT (1998). Enhancing motivation for change in treatment-resistant eating disorders. Clin Psych Rev.

[19] Hay P, Chinn D, Forbes D, Madden S, Newton R, Sugenor L (2014). Royal Australian and new Zealand College of Psychiatrists clinical practice guidelines for the treatment of eating disorders. Aust New Zea J Psych.

[20] Hay P (2013). A systematic review of evidence for psychological treatments in eating disorders: 2005–2012. Int J Eat Disord.

[21] Hilbert A, Hoek HW, Schmidt R (2017). Evidence-based clinical guidelines for eating disorders: international comparison. Curr Op Psych.

[22] National Collaborating Centre for Mental Health (2004). National Institute for Health and Clinical Excellence: Guidance. Eating disorders: core interventions in the treatment and management of anorexia nervosa, bulimia nervosa and related eating disorders.

[23] Murphy R, Straebler S, Cooper Z, Fairburn CG (2010). Cognitive behavioral therapy for eating disorders. Psychiatr Clin North Am.

[24] Bulik CM, Thornton L, Pinheiro AP, Plotnicov K, Klump KL, Brandt H (2008). Suicide attempts in anorexia nervosa. Psych Med.

[25] Rowe E (2017). Early detection of eating disorders in general practice. Aust Fam Phys.

[26] Waller G (2016). Treatment protocols for eating disorders: Clinicians' attitudes, concerns, adherence and difficulties delivering evidence-based psychological interventions. Curr Psych Rep.

[27] Kosmerly S, Waller G, Lafrance RA (2015). Clinician adherence to guidelines in the delivery of family-based therapy for eating disorders. Int J of Eat Disord.

[28] Beidas RS, Kendall PC (2010). Training therapists in evidence-based practice: A critical review of studies from a systems-contextual perspective. Clin Psychol. (New York).

[29] Barrett J, Gonsalvez C, Shires A (2019). Evidence based practice within supervision during psychology practitioner training: a systematic review. Clin Psychol.

[30] Pellizzer ML, Waller G, Wade TD (2019). A pragmatic effectiveness study of 10-session cognitive behavioural therapy (CBT-T) for eating disorders: targeting barriers to treatment provision. Eur Eat Disord Rev.

